# Integrating behavioral assessment in instructional design for competency-based medical education

**DOI:** 10.3389/fmed.2024.1432319

**Published:** 2024-08-16

**Authors:** K. N. Williams, Elizabeth H. Lazzara, M. Sadighi, N. Chandran, K. Joshi, S. Raj, I. Shields, B. Nichols, D. Testa, J. Hernandez, M. Michael, R. Rege, P. Greilich

**Affiliations:** ^1^Department of Human Factors and Behavioral Neurobiology, Embry-Riddle Aeronautical University, Daytona Beach, FL, United States; ^2^Office of Undergraduate Medical Education, UT Southwestern Medical Center, Dallas, TX, United States; ^3^Department of Anesthesiology and Pain Management, UT Southwestern Medical Center, Dallas, TX, United States; ^4^Department of Emergency Medicine, UT Southwestern Medical Center, Dallas, TX, United States; ^5^Department of Anesthesiology and Critical Care, Penn Medicine, Philadelphia, PA, United States; ^6^Department of Pediatrics, UT Southwestern Medical Center, Dallas, TX, United States; ^7^Department of Surgery, Office of Undergraduate Medical Education, UT Southwestern Medical Center, Dallas, TX, United States; ^8^Department of Anesthesiology and Pain Management, Office of Undergraduate Medical Education, Health System Chief Quality Office, UT Southwestern Medical Center, Dallas, TX, United States

**Keywords:** competency-based, undergraduate, medical education, assessment, rater selection, observational ratings

## Abstract

As institutions continuously strive to align with the standards set forth within competency-based medical education, there is an increased need to produce evidence of learner achievement in the form of observable behaviors. However, the complexity of healthcare education and clinical environments make it challenging to generate valid and reliable behavioral assessments. In this article, we utilize our interdisciplinary knowledge from the perspectives of experts in medical education, assessment, and academic administration to provide tips to successfully incorporate behavioral assessments into instructional designs. These include tips for identifying the best assessment methods fit for purpose, guiding instructors in establishing boundaries of assessment, managing instructors, selecting raters, generating behavioral assessment guides, training raters, ensuring logistics support assessment strategies, and fostering capacity for iteration. These can be used by institutions to improve planning and implementation for longitudinal behavioral assessments.

## Introduction

Competency-based assessment is becoming increasingly prevalent in medical education and is vital to meeting goals established in competency-based medical education (CBME) to ensure healthcare workers are adequately prepared to enter the workforce (1). CBME shifts the focus from time-based learning (e.g., a certain number of weeks in each rotation), to a progression-based method that is driven by each learners’ successful demonstration of all relevant competencies (2–4). This is meant to ensure that learners are ready to enter the workforce with all relevant abilities, rather than either assuming they have been achieved based on exposure to content or permitting excellence in some to make up for deficiencies in others.

Some often-overlooked competencies important to clinical performance in all healthcare professions are based in non-technical teamwork and communication (5). Although the impact of technical skills on patient status are often directly and clearly observable (e.g., an incorrect diagnosis will likely lead to inappropriate treatment and lack of improvement in the patient), the impact of non-technical skills such as communication may be less directly tied to patient status (e.g., failure to use closed-loop communication may or may not affect patient care depending on whether an error occurred, and/or was caught via other mechanisms before impacting the patient). Establishing and assessing progression to mastery in such non-technical competencies have been noted as both elusive and highly desirable to adequately meet the goals of CBME (5, 6). Behavioral assessments can provide insights into learner competence in such areas, and their flexibility to be applied to both simulated and clinical settings invite the potential to provide greater insights into mastery over time (6, 7). Behavioral assessments typically consist of observers rating learner performance on sets of discrete behaviors. Examples of technical skills that are often assessed in this context include establishing diagnoses and gaining patient history. In contrast, non-technical skills include behaviors associated with teamwork and communication, such as engaging in interprofessional collaboration, establishing inclusive/psychologically safe environments, and effectively communicating information to appropriate audiences.

Use of simulated settings provides an avenue to increase performance opportunities and reduce theory-practice gaps imposed by learners’ inability to fully participate in patient care while still in training (8). Additionally, they present opportune mechanisms to intentionally create scenarios with progressive difficulty to demonstrate progression to mastery (6). However, there remain a dearth of studies that have successfully linked such simulation activities to performance in practice (i.e., transfer of training), which is greatly hindered by the challenges of implementing longitudinal assessments across these settings (4, 5, 9).

The purpose of this paper is to leverage our experiences in a large academic medical center quality enhancement plan (QEP), which is known as Team FIRST, to offer future educators, practitioners, and researchers some key considerations for incorporating progressive, competency-based, non-technical behavioral performance opportunities within the context of graduate and undergraduate medical education. This manuscript aims to provide prescriptive guidance on how to manage considerations to strengthen the evaluations and provide a framework to effect continual improvement in learners.

## Tip 1

### Not all assessments are equal: consider goals when determining methods, and clearly communicate the boundaries of these to learners, faculty instructors, and stakeholders

There are many different types and purposes of assessments in medical education, which typically range from use of more formative methods typically aimed to understand students’ current abilities for the purpose of providing feedback, to summative methods that are characterized by conferral of a pass or failure score which may be used for competence decisions (10). Oftentimes, the purpose of the assessment becomes ambiguous due to the interests of the stakeholders. This is often particularly true for non-technical skills, given that their impact on patient care is sometimes less readily visible compared to the impact of technical skills. Stakeholders are typically keenly interested in summative assessments that have potential to provide insights regarding the competence of students. Meanwhile, formative assessments are better suited for advancing the competence of learners. Lacking a clear purpose of the assessment may negatively impact both the student acceptance of the assessment, as well as the ability of the assessment to accurately reflect student competence. Heavy use and emphasis of high stakes assessments may reduce students’ willingness to actively engage in those activities due to fear of potential negative scores (10, 11). Furthermore, students may begin to treat success on the assessment as the goal, as opposed to continuous improvement and true mastery in the subject matter, which may cause them to stop pursuing further improvement (10). Lastly, assessments will preclude students from learning when they lack useful, task-oriented feedback (10). These challenges may be alleviated by incorporating smaller, more frequent assessment opportunities that have both summative and formative aspects that are explicitly linked to the learner stage in their development. For instance, Maastricht medical school found success when they integrated formative and summative assessments for strengthening and evaluating professional behaviors (12). Resource costs for such programs may be reduced by incorporating methods for standardized or automatic grading and feedback, though the tradeoffs related to usefulness should be evaluated carefully in the context of overall program goals (13, 14).

## Tip 2

### Do not assume everyone is on the same page: ensure instructor guidance is consistent in implementation relative to assessment elements

Once the format of assessment is determined, it is vitally important to ensure that assessments are implemented and documented consistently. Variations in implementation of such interventions can alter both the ratings achieved and learning outcomes, which impact the validity of the assessment as well as the subsequent interpretation of data corresponding to their progression to mastery (15, 16). For example, having multiple instructors means that some may choose to prompt students, while others may choose to allow failure. Such variations in implementation impact the quality of the behavioral ratings received (17). Variations in ratings can be mitigated by providing scripts or guides that standardize instructors’ introduction of behavioral demonstration opportunities and the expectations associated with them. This is particularly important for non-technical skills, given that behaviors such as closed-loop communication may be liberally or sparsely applied and have potential to impact patient care through either presentation. Thus, establishing and ensuring consistency in expectations for these non-technical competencies is important to ensuring accurate assessment. Vital aspects to emphasize include language specific to assessment (e.g., how assessments may be used), what students should expect of the event (e.g., assignments of specific pairs of partners), common question responses or features of the unspoken curriculum that might otherwise vary across instructors (e.g., expected comprehensiveness of handovers and frequency of closed-loop communication), as well as scripts aimed to enhance student psychological safety (e.g., scripts that emphasize the learning-oriented purpose of assessments and confidentiality where appropriate; 18).

## Tip 3

### Expertise is not everything: consider a variety of perspectives and constraints when selecting raters

There are many individuals who can theoretically serve as raters for behavioral observations. However, there are also a plethora of factors that should be considered when selecting raters. Often at the forefront of these are resource constraints (e.g., financial, scheduling) which are interrelated to other factors that should influence selection, including the respective subject matter expertise and experience of raters. We have described three major types of raters, Novice, Expert/Experienced, and Subject Matter Expert (SME) in Table 1, and described their pros and cons. Standardized Patients (SPs; non-SMEs who participate in simulation sessions and are typically experienced with clinical terminology and rating systems) who have gone through training in behavioral assessment are often timely and reliable resources for evaluation as non-SME, experienced raters (20). Given it is often challenging for SMEs to dedicate sufficient non-clinical time to rate scenarios, their time may be more efficiently utilized in developing grading rubrics and assisting in the training of such dedicated, experienced raters.

**Table 1:** Pros and cons of common observational rater types.

Type descriptions	Pros	Cons
**Novice** *An individual with no experience rating behaviors according to standardized assessments, and no subject matter expertise that is relevant to the domain under study.*	Generally inexpensive to recruit. Scheduling is often flexible. May be less vulnerable to profession-based stereotype effects (i.e., where ratings are based on the learners’ inclusion in a group, such as medical student or nurse, rather than their own unique behaviors; 15).	May be challenging to train and retain (e.g., if the rater does not have sufficient knowledge or commitment related to the goals of the assessment). May not be ideal for obtaining accurate, nuanced, and reliable ratings depending on the purpose of the assessment Case examples of this can be found in the following studies (19–22).
**Expert/Experienced** *An individual with moderate to extensive experience rating behaviors according to standardized assessments (preferably specific ones used in your assessment paradigm), but no subject matter expertise that is relevant to the domain under study.*	Typically, they are less expensive to recruit and retain compared to SMEs. Have demonstrated superiority to novice raters, even when the novice raters were acclimated through basic frame-of-reference training (20).	Often require more resources to recruit and retain compared to novice raters. This type of expertise may not be sufficient to establish reliable and valid ratings, particularly if the assessment is highly domain specific and may require subject matter expertise to effectively differentiate behaviors.
**Subject Matter Expert (SME)** *An individual who has subject matter expertise relevant to the domain under study* (e.g.*, a medical degree*)*, but typically does not have prior experience rating behaviors according to standardized assessments.*	May be more apt at discriminating nuanced behaviors relevant to their domain (21). More readily able to grade the accuracy of behaviors that require domain expertise, such as whether an appropriate diagnosis and treatment was selected, which may be challenging for non-SMEs to recognize even when given extensive training (15).	Typically, they are very expensive to recruit, train, and retain. Schedules are often highly restrictive. Will likely still require training, as evidence demonstrates that even among SMEs, those who are also experienced raters with training in using specific observational tools are more consistent in assessments relative to SMEs who are not experienced raters (19, 22).

## Tip 4

### Even experts need guidance: establish well-defined behaviors and specific examples

Observational ratings are vulnerable to many well-known biases in assessment, including halo (aka horns, i.e., multiple ratings are based on a single observation that is either positive or negative), central tendency (i.e., the tendency to avoid either extreme of ratings), and contrast effects (i.e., ratings are anchored based on the performance of a previous learner or group) among many others (1, 23). Having clear definitions and examples helps to mitigate any ambiguity or confusion while strengthening consistency and accuracy of ratings. Thus, it is important to establish a scoring guide or rubric and a plan for how to rate unclear behaviors (15). To maximize the utility and usability of these scales and guides, faculty subject matter experts and assessment domain experts should collaborate extensively to develop them. It is very helpful to have faculty instructors begin the process of generating this scale in conjunction with assessment experts to develop and refine grading criteria. This helps ensure the rubrics developed have content validity and robustly align to the learning objectives across a multitude of instructors (24).

Guides corresponding to behaviorally anchored rating scales (BARS) can describe the key behaviors and information to be transmitted between learners, such as in the context of simulated handovers. A scale is considered behaviorally-anchored when several levels are present and are tied to specific behaviors that warrant their selection. For example, one of our rating scales addresses whether senders enact behaviors that support a psychologically safe environment. This is behaviorally anchored to response options of (1) “encouraged receiver to ask questions or provide feedback through explicit, open-ended requests (i.e., WHAT questions/feedback do you have),” (2) “made an explicit, but closed-ended, request for questions/ feedback (“DO you have any questions/feedback”), (3) “did not make a request for questions, but allowed space (pause),” and (4) “did not request or allow space (pause) for questions.” This type of response is likely best utilized for behavioral items that have a relatively finite number of discrete options, where divisions can clearly be drawn between response options. Binary scales can be used when behaviors have minimal relevant variation and have clear distinctions between categories (e.g., “sender asked clarifying questions” is unlikely to have substantial meaningful variation outside of a “yes” or “no” response). In situations where there is disagreement about what should be considered a definitive “yes” or “no,” a neutral option (such as “sometime” or “somewhat”) could be included in a binary scale. However, it can be difficult for raters to discriminate between behaviors, especially with numerical ratings (e.g., a 2 vs. 3). Panzarella and Manyon (25) conducted a study in which a diverse team of faculty members worked closely together to create a behavioral tool and its scoring rubric (with clearly defined elements). This tool was designed to evaluate clinical competence in medical and physical therapy programs. In their study, they found the interrater agreement between raters for binary-type responses was significantly higher compared to that observed for a four-point scale. Thus, it is vital to incorporate both subject matter and assessment experts to successfully develop behavioral assessment scales that have sufficient validity and reliability to be used in high stakes assessment contexts.

## Tip 5

### Do not assume guides are enough: train raters thoroughly and hold them accountable for performance

**Figure 1:**
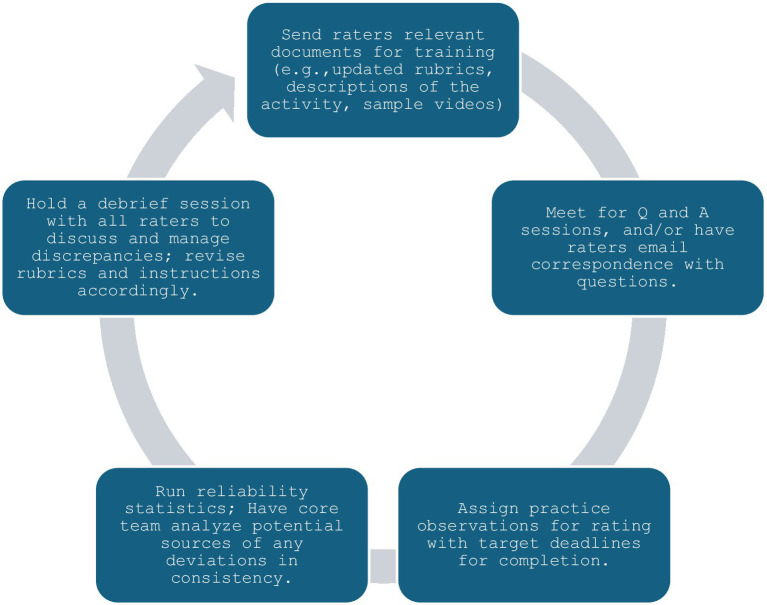
Paradigm for effective rater training to improve reliability.

Many programs will not have the ability to employ a single consistent rater for all instances, which means that multiple individuals will be rating behaviors. To ensure learners are assessed fairly and in ways that support their continual improvement, raters must grade behaviors similarly and consistently with each other and avoid the biases mentioned in the previous section. This is particularly important in the context of CBME, as competence should be unequivocally met or failed based on the student’s ability, regardless of the individual performing any given rating. Development of behavioral instruments and associated rater training has been slower for non-technical skills relative to technical skills, likely due to less shared understanding of concepts associated with non-technical skills (26). This makes targeted training even more important to effectively and consistently assess non-technical skills. Various training methods have been utilized to enhance inter-rater reliability and improve accuracy, including rater error (i.e., inform raters of common mistakes to avoid during grading), performance dimension (i.e., familiarize raters with the assessment dimensions using definitions and descriptions), frame-of-reference (i.e., provide raters with reference cases as their benchmarks, usually examples of poor and good demonstration of the behaviors under assessment, and discuss discrepancies until shared understanding is reached), and behavioral observation training [i.e., target general observation skills to improve anticipation, detection, and grading of behavioral events; 23, 27]. Our group has had success with a training paradigm closely aligned with performance dimension, supplemented by frame-of-reference, using the training schema outlined in Figure 1.

This process can be repeated until acceptable agreement is reached on all assessment items. Ideally, debrief sessions should be held with the entire team. When this cannot occur, sessions should be recorded and provided for those who were absent from the event. If a guide has been crafted (as recommended in the previous tips), modifications can be made using tracked changes with version control, and both clean and tracked change copies sent out to all raters. This helps facilitate all raters recognizing and remaining up to date on grading criteria. Adherence to this process has enabled our QEP Team FIRST program to secure a set of experienced, standardized patient raters who are able to rate over 200 patient handovers using video review in a span of approximately 2 weeks.

## Tip 6

### No matter how well you have planned, it is wise to revisit: ensure the layout and logistics comprehensively support the desired assessment strategy

Administrators and faculty should devote ample resources to the logistical considerations for all phases of events, from conception through assessment, months before planned implementation. Factors that are often pertinent in the event conception phase include determining how groups may be formed and roles selected in interprofessional education sessions. These types of decisions have implications for what can be assessed (e.g., if a teamwork behavior will be observable) as well as how it can be assessed (e.g., if teamwork behaviors are exhibited, are specific behaviors expected between specific roles, etc.), and whether the planned physical space supports the comprehensive and accurate rating of these behaviors. For example, if a small group session necessitates 10 students divide into groups of two to perform handovers to each other simultaneously, live evaluation of this event would require at least five raters, depending on the sequencing of the behaviors to be assessed for each pair.

Additional consideration should be devoted to factors like sound quality in the evaluation space. If an assessment goal requires verbal utterances, it can be very challenging to decipher between learners in a packed room or in video recordings with only room microphones. This is especially difficult with teams, as it is increasingly difficult to decipher who is communicating based on the number of team members present and active in the room. Lapel microphones can be a useful tool for team studies, large rooms, and noisy environments, and devices or software that can identify speakers through name tagging are useful for assessing individual performance. Virtual events can mitigate many of these issues associated with behavioral assessment: When recorded, they can often be utilized to generate transcripts that are attributable to individual speakers, which enable more accurate and nuanced assessment opportunities of individual participants.

## Tip 7

### Development is insufficient: commit to fostering capacity for iteration, innovation, and optimization

To maintain utility, programs must remain responsive to shifts in the needs of the public, organization, incoming learners, and developing learners. Before and after each iteration, the program should be re-evaluated to ensure the same needs exist for learners at baseline, and that the curriculum continues to address these gaps by leading to improvements. It is important to continually monitor for changes to the unspoken curriculum and integrate ways to combat these as needed. Ultimately, programs should maintain awareness of the key competencies desired by national and international institutions, updating the curriculum where needed to maintain relevance. Though this can be challenging given the busy clinical schedules of most educators, it is helpful to have these individuals and assessment experts maintain an excel database of the key competencies and related aspects of curriculum and assessment instruments. This enables straightforward review when competency changes occur, and the related aspects of the curriculum and assessment to be directly targeted to accommodate those changes in a timely manner.

When changes are made, it is beneficial to conduct piloting sessions which include both learners and faculty who have previously experienced the event as well as individuals who have not previously experienced the event. This practice enables those who are experienced to provide feedback on the changes and presents an opportunity to ensure that the content is still cohesively educational for new individuals. It is also important to consider how the structure of the curriculum and assessment may be shifted over time, such as by onboarding new aspects of the existing curriculum that had not previously been considered to further reinforce education and assessment practices (e.g., adding opportunities for assessment and constructive feedback through individual clinical clerkships where possible and appropriate).

## Discussion

As institutions continue to advance assessment to conform to competency-based medical education, they must effectively generate and implement behavioral assessments in their curriculums. To be effective, these must be capable of being longitudinally assessed, clearly documented, and ultimately defensible (i.e., demonstrated as both reliable and valid) to be ethically usable in the context of advancement across stages of CBME. Such assessments have been extensively challenging to attain in terms of both feasibility as well as reliability and validity (5). In this article, we have presented seven tips informed by the literature along with our experiences collaborating as administrative professionals, healthcare providers, medical educators, and experts in assessment which contribute to achieving these goals. These tips are summarized in Table 2, which additionally provides a list of questions that are helpful for staff to complete when integrating these tips.

**Table 2:** Summary of tips and questions to aid implementation.

Tip #	Brief description	Questions to aid implementation
1	Not all assessments are equal: Consider goals when determining methods, and clearly communicate the boundaries of these to learners, faculty instructors, and stakeholders.	What do stakeholders wish to see in learners related to the curriculum? Is there a preference toward knowledge, attitudes, or behavioral demonstration? What elements of this relate most directly to program assessment and funding opportunities? What are the current attitudes of learners, faculty, and key stakeholders related to assessment? How might assessment or communication practices change to accommodate these attitudes and further promote acceptability? What existing assessments relate to the curriculum learning objectives? How might these be leveraged to reduce assessment burden?
2	Do not assume everyone is on the same page: Ensure instructor guidance is consistent in implementation relative to assessment elements.	What unspoken curriculum exists at the institution? Where might this support or conflict with the key learning objectives of the program? Are there specific faculty who may be strongly influencing unspoken curriculum that is detrimental to learning and performance? If so, consider whether additional training, coaching, or reduced exposure to these faculty may limit threats to the learners’ achievement of the learning objectives. Are there clear instructions for facilitators guiding the learners to ensure the assessments can be consistently applied?
3	Expertise is not everything: Consider a variety of perspectives and constraints when selecting raters.	Does assessment of the key learning objectives require subject matter experts or experienced raters? Do faculty have sufficient time to conduct assessments? Do they have sufficient time to dedicate to training for reliability? What resources are available to compensate evaluators for their time conducting assessments?
4	Even experts need guidance: Establish well-defined behaviors and specific examples.	What are the ways learners can respond within the assessment environment? Are there a relatively low number of predictable response types, or are there a wide variety of possible responses? Has the most ideal rater type been selected to perform the assessment? If not, how might more ideal individuals’ experience be leveraged in a more limited capacity to generate guidelines for those who will be performing the evaluations?
5	Do not assume guides are enough: Train raters thoroughly and hold them accountable for performance.	What are the ideal levels of agreement stakeholders expect evaluators to reach for inter-rater reliability? What are the lowest levels acceptable? How do training sessions need to be held to maximize attendance and responsiveness? Are in-person meetings appropriate, or are virtual and/or asynchronous meetings sufficient? How much time can be dedicated to training? Are there sufficient resources to meet with evaluators on a regular basis during the times they are available (e.g., are experienced training staff available after business hours if needed)? What additional resources might be needed to accommodate these schedules (e.g., additional hires, overtime pay, flexible work hour arrangements, etc.)?
6	No matter how well you have planned, it is wise to revisit: Ensure the layout and logistics comprehensively support the desired assessment strategy.	Are evaluators rating based off live performance or video review? Do assessment forms support completion in this environment? Are sufficient tools available to support learner identification? What is the ratio of learners to evaluators? Is this adequate for the assessment environment? What interprofessional differences are there in competency demonstration, and has this been incorporated into assessment plans? If applicable, what are plans to share assessment data across professional schools?
7	Development is insufficient: Commit to fostering capacity for iteration, innovation, and optimization.	How are the baseline KSAs of students changing upon entry from cohort to cohort? How has the unspoken curriculum developed over time? What changes are needed to accommodate the needs of new learners and new environments? How often do competencies change at the national or international level? What staff might be available to maintain a database of linkages between the competencies, curriculum and assessment instruments?

Tip 1 emphasizes the importance of aligning assessment methods with specific goals and clearly communicating these to all stakeholders. By differentiating between formative and summative assessments and incorporating both aspects into the evaluation process, educators can foster a culture of continuous improvement while ensuring accurate assessments of student competence. This tip may be most simply implemented by utilizing frequent assessments wherein findings communicated to the learner are formative and anchored to the learners’ current stage of development, and only summed after repeated instances of observation (e.g., learners may be formatively assessed monthly or quarterly, and this information summed yearly for advancement purposes). Identifying ways to focus on the developmental progression of the learner over time may facilitate building growth-oriented mindsets in learners and assessors, potentially reducing current barriers perceived in the transitions from UME to GME space (28).

Tip 2 outlines strategies related to consistency in assessment implementation to maintain the validity and reliability of evaluation outcomes. Standardizing instructor guidance and providing clear expectations for both students and instructors can mitigate variations in assessment practices and enhance the quality of behavioral ratings. This can most effectively be addressed by providing instructors with cheat sheets to aid their consistency and performing periodic quality checks on sessions via in person, video, or assessment score review to ensure facilitators are being consistent in their messaging. Facilitators not adhering to the appropriate messaging may be identified by comparing student assessment scores across facilitators, and targeting the faculty with the highest and lowest performing students overall to determine if their scores relate to differences in facilitator guidance.

Tip 3 highlights the necessity of considering various perspectives and constraints when selecting raters for behavioral observations. By identifying different types of raters and their respective advantages and limitations, educators can strategically allocate resources and optimize the reliability and validity of assessment results within their institution. For example, an assessment geared toward assessing nuanced non-technical skills that are unrelated to technical skills (e.g., utilizing behaviors that establish a psychologically safe environment) is likely to be an appropriate target for a novice or experienced rater, given these individuals have time to participate in more extensive training. In contrast, an assessment geared toward assessing non-technical skills that overlap with technical skills (e.g., utilizing a handover protocol to more effectively communicate patient information or utilizing closed-loop communication) may require a subject matter expert rater to ensure that non-technical aspects are appropriately paired with technical behaviors that are not the primary target, but relate to assessment.

Tip 4 emphasizes the importance of establishing well-defined behaviors and specific examples to mitigate biases and enhance the accuracy of ratings. For assessment items where response options are relatively finite, behaviorally anchored rating scales and/or checklists are likely to be most appropriate. In contrast, assessment items where infinite types of responses are possible may be more appropriate targets for quality-based numerical scales. For example, to assess handover quality in a clinical environment, an item asking the observer to rate “How effective was the handover overall?” on a 5-point Likert type scale from “not effective at all” to “extremely effective” may be appropriate. However, such an item would likely achieve more accurate and reliable ratings from experienced subject matter experts, as they have a more practical experience base to draw from to assess handover “effectiveness” compared to raters who have never participated in handovers.

All observational assessments and raters should be verified through rater training: Accordingly, Tip 5 reflects the importance of rater training to improve inter-rater reliability and accuracy in behavioral assessments. Rater training should consider both the type of rater being used as well as the type of assessment items to ensure maximum improvement in reliability. Paradigms such as error training and performance dimension may be most effective for novice/experienced raters using behaviorally-anchored or checklist-based scales, whereas paradigms such as frame-of-reference and observational training may be more effective for subject matter experts utilizing numerical rating scales. This recommendation relates to our earlier discussion of the responses for these respective items: Whereas behaviorally-anchored and checklist-based items have clearly observable distinctions between responses that lend themselves to explicit corrections using error and performance-dimension training, numerical rating/Likert-type scales typically do not have as clear distinctions between response options and are therefore better candidates for calibration (instead of correction) using frame-of-reference and observational training. These training paradigms (frame-of-reference and observational) may also be particularly helpful for training related to assessments that occur in the clinical environment, given that there is a wide range of environments the observer may assess the learner in for any given competencies.

Tip 6 underscores the significance of logistical considerations in supporting the desired assessment strategy. The most comprehensive way to implement this tip is to establish a thorough infrastructure to generate and store video recordings of assessment occurrences. This enables continuous nuanced assessment and more widespread utility for assessment instances that is useful for collaboration between professional schools.

Tip 7 reflects the need for continuous improvement in the curriculum based on changes that will inevitably occur during a longitudinal program with repeated implementation across cohorts. Changes should not be sporadic but should be appropriately responsive to the changes that occur both within and outside the program. This process can be greatly assisted by maintaining a database of key competencies, related curriculum and assessment elements to ensure targeted evaluation of changes and corresponding corrections to the most relevant aspects of the program.

Successful implementation and sustainment of longitudinal behavioral assessments in the context of CBME is challenging, but we hope these tips will help other institutions more effectively navigate this process.

## Data availability statement

The original contributions presented in the study are included in the article/supplementary material, further inquiries can be directed to the corresponding author.

## Author contributions

KW: Conceptualization, Investigation, Project administration, Writing – original draft, Writing – review & editing. EL: Conceptualization, Investigation, Writing – original draft, Writing – review & editing. MS: Conceptualization, Investigation, Project administration, Writing – original draft, Writing – review & editing. NC: Investigation, Writing – original draft, Writing – review & editing. KJ: Writing – review & editing. SR: Writing – review & editing. IS: Writing – review & editing. BN: Writing – review & editing. DT: Writing – review & editing. JH: Writing – review & editing. MM: Writing – review & editing. RR: Writing – review & editing. PG: Writing – review & editing.
